# Seasonal dynamics of *Galaxea fascicularis* holobiont from physiological to transcriptional responses and implications for natural resilience

**DOI:** 10.3389/fmicb.2025.1707108

**Published:** 2025-12-19

**Authors:** Yushan Li, Jingzhao Ke, Haiyan Yang, Xiangbo Liu, Junling Zhang, Mingce Huangfu, Jinling Liu, Wentao Zhu, Aimin Wang, Rou-Wen Chen, Xiubao Li

**Affiliations:** 1 Key Laboratory of Tropical Hydrobiology and Biotechnology of Hainan Province, School of Marine Biology and Fisheries, Hainan University, Haikou, China; 2 Wenchang Advanced Fisheries Research Institute, Hainan University, Wenchang, China; 3 International Joint Research Center for Coral Reef Ecology of Hainan Province, Hainan University, Haikou, China

**Keywords:** coral holobiont, *Galaxea fascicularis*, natural resilience, physiology, seasonal variation, transcriptome

## Abstract

Monitoring seasonal changes in coral holobionts throughout the year is essential for understanding coral resilience and symbiotic responses. Previous studies have focused on short-term or specific seasonal changes, limiting their ability to capture annual variations. This study on *Galaxea fascicularis* in the South China Sea integrates physiological, symbiotic, and transcriptomic analyses across all seasons. In spring, upregulation of Symbiodiniaceae photosynthetic genes and lipid synthesis genes enhances coral photosynthesis and lipid accumulation, promoting growth and reproduction. During July–September, seawater temperatures at the Wuzhizhou Island approached the coral bleaching alert level 2. Summer heat stress reduced photosynthetic capacity, shifted corals to heterotrophy (Δ^h-z 13^C < 0), and increased MDA content threefold. Signaling pathways, antioxidant systems, and immune pathways were activated. Coral recovery began in autumn and winter after the summer heat and reproduction. In autumn, autotrophy increased, and immunity was activated to repair oxidative damage. In winter, processes for skeleton growth, energy storage, and metabolism were enhanced. Endosymbiotic *Durusidinium* remained stable, while *Endozoicomonas* abundance decreased in summer. In winter, potential pathogenic bacteria like *Acinetobacter* increased. These findings highlight the coral holobiont’s synergistic response to seasonal changes, validating coral resilience and guiding artificial restoration strategies.

## 1 Introduction

Coral growth is crucial for sustaining coral reef ecosystems and facilitating reef development. However, in recent decades, fringing reefs in the low-latitude tropical region of the South China Sea (SCS) have experienced severe bleaching and mortality events in 2015, 2016, and 2022 (Yu, 2012; Goyen et al., 2019). In the face of this critical situation, artificially assisted restoration strategies have emerged as a crucial approach to safeguard and revive these delicate ecosystems (Caruso et al., 2021). Successful coral transplantation relies heavily on pre-transplantation assessment of the natural resilience of target species, as it directly determines the survival rate of transplanted corals and the overall success of restoration projects. *Galaxea fascicularis*, known for its relatively high tolerance, holds significant potential in coral reef restoration. However, a limited understanding of the seasonal adaptive mechanisms underlying resilience in *G. fascicularis*, specifically the synergistic physiological and transcriptional responses within its coral holobiont (host, endosymbiotic microalgae, and associated bacteria), hinders the optimization of restoration strategies.

Seasonal environmental fluctuations drive dynamic changes in coral holobiont function. In spring, warmer seawater promotes the growth of both coral hosts and their symbionts. Furthermore, healthy endosymbiotic microalgae transfer photosynthetic metabolites that provide energy and nutrients to corals while promoting their growth, reproduction, and calcification processes (Fransolet et al., 2012; Torres et al., 2021). Annual coral spawning events display distinct seasonal patterns, and numerous coral species engage in spawning and reproduction in the spring (Bouwmeester et al., 2015; Wei et al., 2023). Lipids, being a significant source of energy for coral reproduction, accumulate during oocyte maturation until spawning (Oku et al., 2003; Lin et al., 2012). Corals face various environmental challenges throughout the year. The high temperatures in summer may trigger heat stress responses, leading to a decrease in symbiotic microalgal density, chlorophyll a content, and photosynthetic capacity (Vajed Samiei et al., 2015). In this case, corals may produce harmful reactive oxygen species, leading to lipid peroxidation damage and affecting the immunity, repair, and cell growth of the coral host, and in severe cases, even triggering coral bleaching and death (Lushchak, 2011; Jia et al., 2024). In addition to possible thermal perturbation in summer, a decline in seawater temperature in winter may lead to a corresponding alteration within microbial communities (Yu et al., 2023). When opportunistic and potentially pathogenic bacteria are present, they might cause an increased occurrence of coral diseases (Gignoux-Wolfsohn et al., 2017). Despite these known seasonal challenges, most previous studies have focused on short-term stress responses or single-season observations (e.g., summer heat stress alone), failing to capture the continuous annual dynamics of the coral holobiont (Li et al., 2021b; Thomas et al., 2019). This knowledge gap hinders our understanding of the long-term adaptive strategies that underpin coral resilience and limits the translation of basic research to applied restoration.

Coral seasonal adaptation is not driven by the host alone but by the joint regulation of the entire holobiont. For instance, *G. fascicularis* acquires energy through both heterotrophic predation and photosynthates from endosymbiotic microalgae (Xu et al., 2022; Fransolet et al., 2012; Torres et al., 2021), and can adjust symbiont communities or rely on host energy reserves under stress (Strychar and Sammarco, 2009; Silverstein et al., 2015; Li et al., 2021b). At the transcriptional level, coral hosts and endosymbiotic microalgae have different gene expression patterns and cell signaling pathways. For example, heat stress upregulates apoptosis and immunity pathways in hosts and calcium/cAMP signaling in microalgae (Li et al., 2021b). Symbiotic bacteria further contribute to energy supply, nutrient cycling, and immune regulation, playing key roles in post-bleaching recovery (Pernice et al., 2020; Thomas et al., 2019). However, existing studies rarely integrate physiological, symbiotic, and transcriptional data to dissect the holistic adaptive mechanisms of the *G. fascicularis* holobiont across seasons. Merely focusing on individual physiological traits (e.g., bleaching indicators or energy reserves) fails to reveal the genetic underpinnings of seasonal adaptation or the synergistic interactions among holobiont components, creating a critical disconnect between basic resilience research and applied restoration needs.

To address these gaps, we hypothesize that *G. fascicularis* achieves seasonal adaptation through holobiont synergistic regulation, with coordinated adjustments in trophic strategy, symbiont community structure, and gene expression profiles in response to seasonal environmental fluctuations. We focused on the following research questions: (1) How does the physiology of *G. fascicularis* and its symbionts vary seasonally? (2) What temporal transcriptional mechanisms underpin the seasonal adaptation of the coral host and endosymbiotic microalgae? (3) What are the respective roles of the host, Symbiodiniaceae, and associated bacteria in seasonal adaptation? To answer these, we conducted a 1-year integrated study (April 2023–January 2024) of *G. fascicularis* at Wuzhizhou Island (WZZ), SCS, combining environmental monitoring, physiological assays, and multi-omics analyses (ITS sequencing for microalgae, 16S rRNA sequencing for bacteria, and RNA-seq for host and microalgae). This study aims to comprehensively elucidate the seasonal adaptive mechanisms of the *G. fascicularis* holobiont, filling the knowledge gap between short-term stress responses and long-term resilience. The findings will not only advance our understanding of coral holobiont dynamics under natural seasonal fluctuations but also provide mechanistic insights for optimizing coral transplantation timing and selecting resilient corals, ultimately enhancing coral reef restoration effectiveness in the SCS.

## 2 Materials and methods

### 2.1 Study area and sample collection

The study site was located at a depth of 3–4 m on WZZ Island, Sanya, China (18° 19.031′N, 109° 46.123′E). Divers labeled numbered cards on five healthy *Galaxea fascicularis* colonies by SCUBA diving, ensuring that the same individual colonies were consistently resampled in each season (Supplementary Figure S1). *G. fascicularis* was collected and identified through morphology and molecular methods (Supplementary Figure S2; Supplementary Table S1). Coral samples were located within 2 m of each other to ensure spatial consistency. Each sample with a diameter of about 10 cm was collected during spring (22 April, 2023, *n* = 5), summer (8 July, 2023, *n* = 5), autumn (22 October, 2023, *n* = 5), and winter (8 January, 2024, *n* = 5). Sample collection was permitted by the Department of Agriculture and Rural Affairs of Hainan Province. The state of each sample in various seasons was documented using a TG–6 waterproof camera. The collected corals were temporarily kept in oxygenated containers, and photosynthetic capacity parameters were determined *in situ* on live coral samples using a MINI-PAM-II fluorometer while their tentacles were fully stretched. Subsequently, the live coral samples were divided into two portions for laboratory experiments: half were immediately frozen in liquid nitrogen (−80 °C) for molecular assays, and the remainder were stored at −20 °C for laboratory-based physiological and biochemical analyses.

### 2.2 Seasonal seawater characteristics data

A HOBO thermometer (ONSET, USA, UA-002-64) was placed adjacent to the labeled *G. fascicularis* colonies for the first time (22 April, 2023) to monitor real-time seawater temperature (°C) throughout the annual period. Turbidity (FTU) was assessed using an AQUAlogger 210 (Aquatec, UK). Other seawater characteristics, such as seawater temperature (°C), salinity (PSU), pH, and dissolved oxygen (mg/L), were measured *in situ* with a water quality multiprobe (Eureka Water Probes, USA). All parameters were measured in spring (22 April, 2023), summer (8 July 2023), autumn (22 October, 2023), and winter (8 January, 2024), and they were measured 15 times.

### 2.3 Determination of stable carbon isotope (δ^13^C)

Samples of *G. fascicularis* from different seasons were pre-treated following the method described by Li et al. (2022). The carbon isotope of the host tissue and endosymbiotic microalgae were prepared by sonication using an ultrasonic crusher (SCIENTZ-950E), followed by super-centrifugation at different speeds (400 r/min and 4,000 r/min) and times (5 min, 10 min, and 30 min). The host tissue and endosymbiotic microalgae were completely separated.

The carbon isotope values of the host tissue (δ^13^C_h_) and endosymbiotic microalgae (δ^13^C_z_) were analyzed using a Delta Plus XP isotope ratio mass spectrometer, with measurements expressed in ‰. Additionally, the contribution of coral heterotrophy relative to autotrophy was assessed by calculating Δ^h-z 13^C (Muscatine et al., 1989). When the absolute values of δ^13^C_h_ exceed the absolute values of δ^13^C_z_, it indicates a greater contribution of heterotrophy. Conversely, autotrophy contributes more to organic carbon fixation. Therefore, a smaller difference in Δ^h-z 13^C suggests a more heterotrophy for the corals (Schoepf et al., 2015).

### 2.4 Photosynthetic capacity, endosymbiotic microalgal density, and chlorophyll a content

Following a 30-min dark reaction of live coral samples, the chlorophyll-modulated fluorometer MINI-PAM-II (Walz, Germany) was used to non-invasively measure the maximum quantum yield (Fv/Fm) of photosystem II (PSII) in the endosymbiotic microalgae. Afterward, live coral samples were exposed to light. Once the values reached a stable state, selecting the “Act. L” function maintained actinic light, and a saturation pulse was triggered via the “SAT” button to determine Y(II). In addition, we also measured *α* (the initial slope, mol electron per mol photon), which reflected the efficiency of light energy utilization by the endosymbiotic microalgae. All parameters were assessed at five randomly selected locations within each coral sample.

The endosymbiotic microalgal density was determined as previously described (Zhu et al., 2021). The coral tissue was rinsed with a Waterpik dental scaler (Waterpik) containing filtered seawater (0.45 μm, Whatman, UK), and the endosymbiotic microalgal density was determined using a hemocytometer under a biological microscope (OLYMPUS, XC23). Each sample was counted eight times, and the average value was calculated. Chlorophyll a (Chl a) content (mg/g) was determined using a microplate assay kit (AIDISHENG Biotechnology, China, ADS–W–GH008) according to the manufacturer’s instructions. Each coral sample was measured three times.

### 2.5 Coral stress and energy characteristics

Malondialdehyde (MDA), Caspase 3, and total antioxidant capacity (T–AOC) were used to determine the extent of oxidative damage to *G. fascicularis*. MDA (nmol/mg prot) and Caspase 3 (U/mg prot) were measured according to the manufacturer’s kit method (Grace Biotechnology, China).^1^ T-AOC (mmol/g prot) was measured according to the kit instructions (Nanjing Jiancheng Bioengineering Institute, China).

The total protein content (mg cm^−2^) in coral homogenates was quantified by a modified bicinchoninic acid protein assay kit (SANGON Biotech, China). Carbohydrates (mg cm^−2^) were determined by the phenol–sulfuric acid method (DuBois et al., 1956). Total lipids (mg cm^−2^) were determined according to the method of Grottoli et al. (2004). The coral lipids were extracted using the solution (V_Methanol_: V_Chloroform_ = 1:2). Then, one-fifth of the solution volume of 0.88% KCl was added, followed by dark treatment for extraction for about 24 h. Subsequently, the lipid extract was placed in 39 °C N_2_ gas vacuum to evaporate and dry.

### 2.6 DNA extraction, sequencing, and data analyses

The total genomic DNA was extracted from 20 samples using the DNA Extraction Kit for Marine Animal Genome (TIANGEN, Beijing, China) following the manufacturer’s instructions. The primers 188–1 (5’-GAATAGGGTATACTAGCAGGTC-3′) and 188-R2 (5′-TTTGCCTTTCCGTATCCACCAT-3′) (Watanabe et al., 2005) were used to identify the mitochondrial type of the non-coding region between *cyt* b and *nad* 2 (Supplementary Table S1). The ITS2 region of Symbiodiniaceae was amplified using primers GTL2-F (5’-GAATTGCAGAACTCCGTG-3′) and GTL2-R (5’-GGGATCCATATGCTTAAGTTCAGCGGGT-3′) (Lajeunesse and Trench, 2000). Bacterial 16S rRNA V3–V4 was amplified by PCR using bacterial universal primers 338F (5′-ACTCCTACGGGAGGCAGCA-3′) and 806R (5′-GGACTACHVGGGTWTCTAAT-3′; Huse et al., 2008). PCR was amplified and purified using previously described methods (Chen et al., 2024). PCR amplification was conducted in triplicate, and the corresponding PCR products were pooled for each sample. The purified amplicons were pooled in equimolar concentrations and subsequently paired-end sequenced (2 × 300 bp) using the Illumina MiSeq PE300 platform (Illumina, San Diego, CA, USA), according to the standard protocols of Majorbio Bio–pharm technology (Shanghai, China).

The original sequencing reads were demultiplexed, quality-filtered by Fastp version 0.20.0 (Chen et al., 2018), and merged by FLASH version 1.2.7 (Magoč and Salzberg, 2011). The optimized data were then processed using the Divisive Amplicon Denoising Algorithm-2 (DADA2) (Callahan et al., 2016) to obtain the Amplicon Sequence Variant (ASV) representing sequence and abundance information. For taxonomic classification, the Sym–ITS2 database,^2^ which provided access to the 621 core and the 750 additional distinct ITS2 variants, was derived from 2,739 high-quality Symbiodiniaceae ITS2 sequences (Shi et al., 2021). Each Symbiodiniaceae ASV was annotated against the Sym–ITS2 with a threshold of e-value 10^−5^ relative to the most similar clade hit. The bacterial ASV representative sequence was analyzed by RDP Classifier (Wang et al., 2007), and each ASV representative was annotated against the 16S rRNA database (Silva v138).

### 2.7 RNA extraction, sequencing, and data analyses

Total RNA was extracted from coral samples using TRIzol® Reagent according to the manufacturer’s instructions. Isolated RNA was dissolved in RNase-free water and stored at −80 °C. RNA quality and quantity were assessed using electrophoretic profiling, performed on 5,300 Bioanalyser (Agilent Technologies, Santa Clara, CA, USA). RNA purification, reverse transcription, library construction, and sequencing were performed at Majorbio Bio-Pharm Technology (Shanghai, China). The paired-end RNA-seq sequencing library was sequenced with the NovaSeq X Plus sequencer. After quality filtration, the clean reads of each sample were separately aligned to the designated reference genome using HISAT2 software (Kim et al., 2015). The reference genome was *Galaxea fascicularis* (gfas_1.0),^3^ and for the Symbiodiniaceae, it was *Durusdinium trenchii* (GenBank: GCA_963970005.1).

Differentially expressed genes (DEGs) were screened by pairwise season comparisons using the DESeq2 R package (version 1.40.2), and the DEGs profiles for each season were built (FC ≥ 2 and FC ≤ 0.5, *P*-adjust < 0.05, Bonferroni). Each season was further clustered based on temporal expression patterns using the Short Time-series Expression Miner (STEM) software (version 1.3.11) to produce 20 DEGs clusters (*R* > 0.7, *p* < 0.05). Furthermore, the significantly upregulated or downregulated gene clusters were further selected and merged, respectively. The merged DEG sets were validated using the Mfuzz R package (version 2.60.0),^4^ and default parameters were used. Finally, the representative DEGs sets were annotated against the databases (NR, KEGG, EggNOG, Pfam, and Swissprot) and subsequently subjected to functional annotation. Enrichment analysis of KEGG pathways was conducted using the clusterProfiler R package (version 4.8.2), with a significance threshold of *p*-adjust < 0.05.

### 2.8 Other statistical analyses

All statistical calculations were performed using IBM SPSS Statistics (version 27.0, Released 2020, IBM Corp., Armonk, New York, United States). Photosynthesis data and physiological analysis conformed to normality and homogeneity, and they were analyzed by one-way ANOVA with *post hoc* Tukey–HSD test. The seawater characteristics were non-normal, so the comparison of seawater characteristics among seasons was performed by the Kruskal–Wallis test. Significance was marked by different letters. All data are presented as mean ± standard deviation.

Non-metric multidimensional scaling (NMDS) based on Bray–Curtis distances was performed to investigate coral symbionts (Symbiodiniaceae and bacterial communities) in four seasons. The statistical significance was evaluated using an analysis of similarities (ANOSIM). Permutation multivariate analysis of variance (PERMANOVA) was employed to assess potential significant differences with default settings (Bray–Curtis distance and 999 permutations). Linear discriminant analysis effect size (LEfSe, LDA scores = 4) was used to determine further whether specific individual bacterial taxa were differentially enriched among four seasons, and the statistical significance was evaluated using strict all-against-all analysis (Segata et al., 2011). The potential bacterial functions were predicted by PICRUSt2 and FAPROTAX based on the bacterial classification information. All data were analyzed using the online tool of Majorbio Cloud Platform.^5^ The co-occurrence network was constructed using Gephi (version 0.9.2) and visualized. The correlation coefficient used in the network analysis was Spearman’s correlation (*r*) > 0.6 and had a statistically significant *p* value < 0.05 (Barberán et al., 2012). The topology of the resulting network was characterized by a set of metrics, including node connectivity, average path length, modularity, and correlation.

## 3 Result

### 3.1 Seawater environment characteristics in four seasons

**Figure 1:**
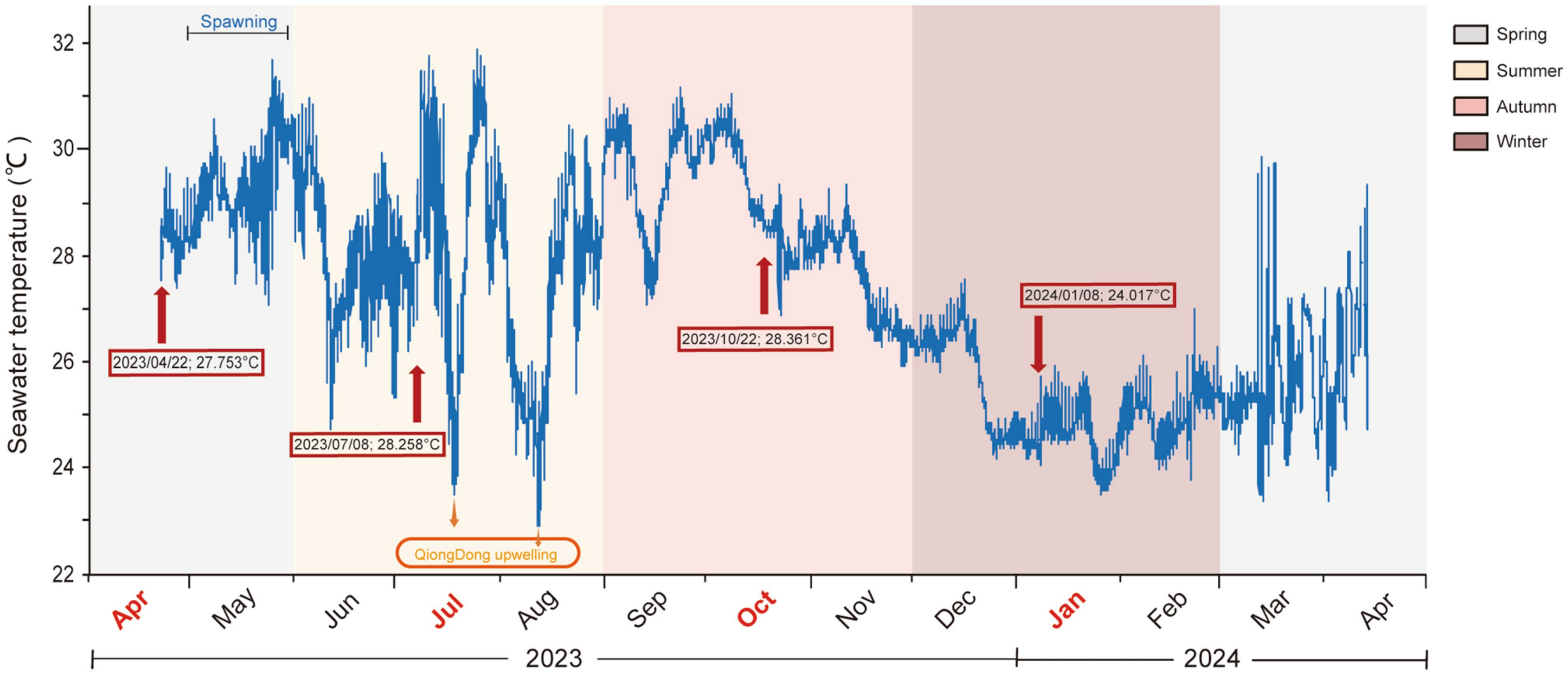
The seawater temperature surrounding samples of *G. fascicularis* on WZZ Island. Seawater temperature was continuously monitored throughout the year on WZZ Island (18°19.031′N, 109°46.123′E). The spawning of *G. fascicularis* occurs in April and May each year, and there were two Qiongdong Upwelling (QDU) events in total that occurred in mid-July and mid-August.

Seawater characteristics were obtained for different seasons in the WZZ. The seawater temperatures ranged from 22.43 °C to 32.17 °C throughout the year (Figure 1). The highest mean seawater temperatures occurred in autumn (Sep–Nov, 28.74 °C ± 0.89), followed by summer (Jun–Aug, 27.88 °C ± 0.55) and spring (Mar–May, 27.73 °C ± 1.61). In contrast, the lowest seawater temperatures were observed in winter (Dec–Feb, 25.15 °C ± 0.53). During July–September, the seawater temperature in the WZZ was close to the coral bleaching alert level 2 (1 ≤ Hotspot and 8 ≤ DHW ≤ 12) in Hainan Island (China), as reported by NOAA Coral Reef Watch.^6^ Nevertheless, there were two Qiongdong Upwelling (QDU, recorded by Wentao Zhu) events with sustained low seawater temperatures in mid-July (23.79 °C ± 0.78) and mid-August (23.38 °C ± 0.11). Overall, the turbidity ranged from 1.17 ± 0.59 FTU to 4.14 ± 0.12 FTU and showed significant highest values in winter (*p* < 0.05). In addition, the highest salinity, pH, and water dissolved oxygen were in summer (averaging 33.60 ± 0.02 PSU, 8.12 ± 0.01, and 7.41 ± 0.02 mg/L, respectively) (Supplementary Table S2, *p* < 0.05, Kruskal–Wallis test).

### 3.2 Stable carbon isotope content

**Figure 2:**
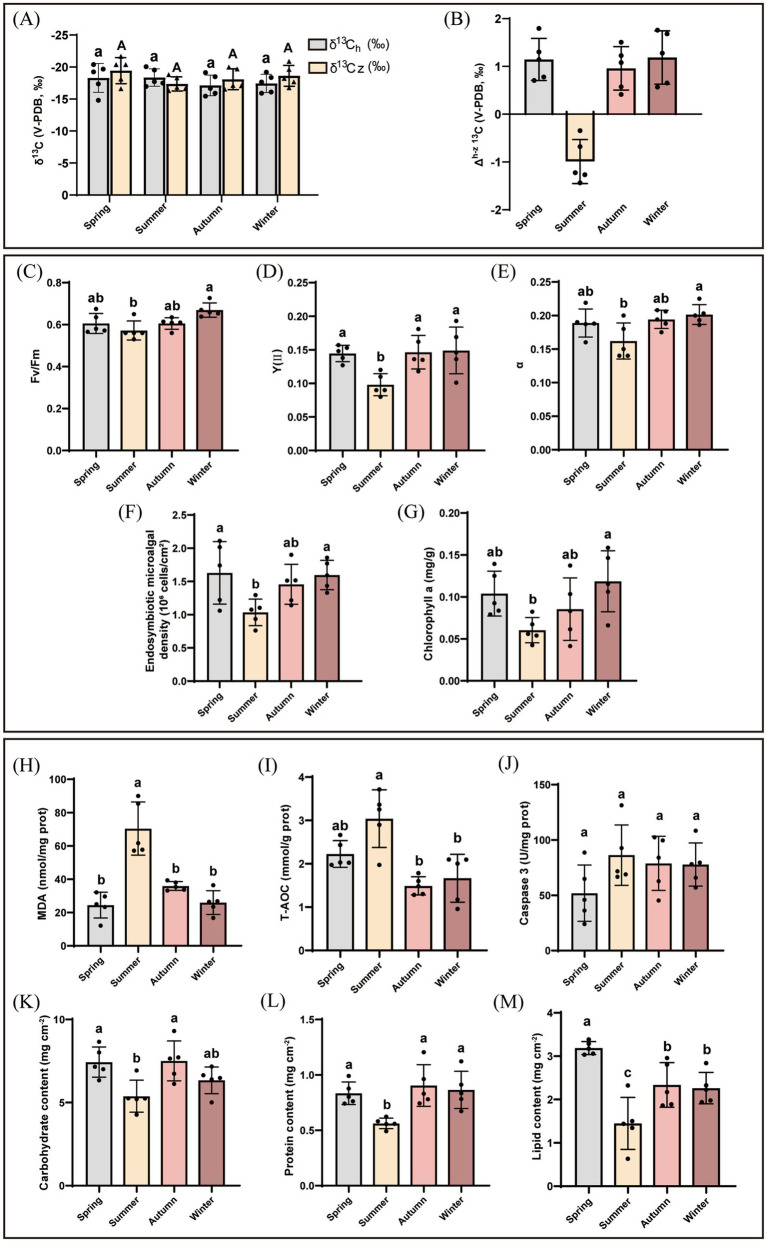
Photosynthetic capacity and energy characteristics of *G. fascicularis* samples. They were measured in spring (*n* = 5), summer (*n* = 5), autumn (*n* = 5), and winter (*n* = 5) on WZZ Island, including **(A)** the average δ^13^C_h_ and δ^13^C_z_ values of coral samples, **(B)** the average Δ^h-z 13^C values of coral samples. Physiological indicators of coral endosymbiotic microalgae include **(C)** Fv/Fm, the potential maximum quantum yield, **(D)** Y(II), the effective PSII quantum yield, **(E)** *α*, the light use efficiency, **(F)** endosymbiotic microalgal density (10^6^ cells/cm^2^), and **(G)** chlorophyll a (mg/g). Each biological parallel sample was measured five times. *G. fascicularis* stress and energy characteristics include **(H)** malondialdehyde (MDA, nmol/mg prot), **(I)** T–AOC (mmol/g prot), **(J)** Caspase 3 (U/mg prot), **(K)** carbohydrate content (mg cm^−2^), **(L)** protein content (mg cm^−2^), and **(M)** lipid content (mg cm^−2^). Each biological parallel sample was measured at least three times. Different letter marks **(A–C)** indicated a significant difference, and the same letter indicated no significant difference.

In spring, autumn, and winter, the absolute values of δ^13^C_h_ were higher than the absolute values of δ^13^C_z_ in *G. fascicularis* (Figure 2A), and the organic carbon fixation Δ^h-z^ δ^13^C > 0 in these three seasons were 1.44 ± 1.90‰, 0.96 ± 1.45‰, and 1.18 ± 1.37‰, respectively (Figure 2B, *P* > 0.05, one–way ANOVA). Conversely, in summer, the δ^13^C_h_ was −18.36 ± 1.21 ‰, the δ^13^C_z_ was −17.37 ± 0.99‰, and the organic carbon fixation Δ^h-z^ δ^13^C < 0 (Figure 2B). Overall, the WZZ population of *G. fascicularis* exhibited a relatively higher photosynthetic autotrophic ability in spring, autumn, and winter, while demonstrating a relatively stronger heterotrophic feeding ability in summer.

### 3.3 Photosynthetic capacity, endosymbiotic microalgal density, and chlorophyll a content

The summer season exhibited significantly lower levels of photosynthetic capacity, endosymbiotic microalgal density, and chlorophyll a compared to other periods (Figures 2C–2G, *P* < 0.05). The average values of Fv/Fm, Y(II), and *α* of *G. fascicularis* were lower in summer (0.57 ± 0.04, 0.11 ± 0.03, 0.16 ± 0.02, respectively) than in spring (0.61 ± 0.04, 0.14 ± 0.01, 0.19 ± 0.02), autumn (0.61 ± 0.02, 0.15 ± 0.02, 0.19 ± 0.01), and winter (0.67 ± 0.03, 0.15 ± 0.03, 0.17 ± 0.05) (Figures 2C–2E). Similarly, the endosymbiotic microalgal density and chlorophyll a in *G. fascicularis* exhibited significant seasonal variations, with the lowest values observed in summer (1.03 ± 0.17 × 10^6^ cells cm^−2^, 0.06 ± 0.02 mg/g, respectively), followed by autumn (1.45 ± 0.27 × 10^6^ cells cm^−2^, 0.09 ± 0.03 mg/g). Conversely, higher endosymbiotic microalgal densities were recorded in spring (1.63 ± 0.42 × 10^6^ cells cm^−2^) and winter (1.60 ± 0.20 × 10^6^ cells cm^−2^), accompanied by increased chlorophyll a content (0.10 ± 0.02 mg/g in spring and 0.12 ± 0.03 mg/g in winter) compared to summer and autumn (Figures 2F,G, *P* < 0.05).

### 3.4 Coral stress and energy characteristics

The coral stress (Figures 2H–J) and energy content (Figures 2K–M) of *G. fascicularis* exhibited seasonal variations, with summer showing significant differences compared to the other seasons. The MDA content in summer (70.39 ± 14.28 nmol/mg prot) was triple as high as that in other seasons, exhibiting a significant increase compared to spring (24.41 ± 6.94 nmol/mg prot), autumn (35.90 ± 2.39 nmol/mg prot), and winter (25.94 ± 6.35 nmol/mg prot) (Figure 2H, *P* < 0 0.05). Similarly, T-AOC content was significantly higher in summer, 3.04 ± 0.60 mmol/mg prot (Figure 2I), compared with the other three seasons (2.28 ± 0.38 mmol/mg prot in spring, 1.50 ± 0.19 mmol/mg prot in autumn, and 2.00 ± 0.88 mmol/mg prot in winter, respectively, *p* < 0.05). The Caspase 3 activity exhibited no statistically significant difference across the seasons (Figure 2J, *P* > 0.05). Compared with the other three seasons, summer exhibited significantly lower values of energy content (*p* < 0.05), with carbohydrate content being 5.38 ± 0.86 mg cm^−2^ (Figure 2K), protein content being 0.56 ± 0.55 mg cm^−2^ (Figure 2L), and lipid content being 1.44 ± 0.54 mg cm^−2^ (Figure 2M). The lipid content in spring was the highest with 3.19 ± 0.13 mg cm^−2^ (Figure 2M, *P* < 0.05), which increased by 2.2 times compared to summer, 1.5 times compared to autumn, and 1.4 times compared to winter.

### 3.5 Symbiodiniaceae community composition

The analysis yielded a total of 2,519,046 high-quality sequences from 20 samples, encompassing 718,933,254 qualified bases. After being rarefied by the lowest number of sequence samples (*n* = 21,749), Symbiodiniaceae ITS2 sequences clustered into 39 ASVs affiliated with *Cladocopium* and *Durusdinium* (Supplementary Table S3). NMDS analyses based on the Bray–Curtis distance (stress = 0, *R* = −0.0202, *p* = 0.537000) and the PERMANOVA result (Supplementary Table S4, *F* = 0.43, *R^2^* = 0.07, *p* = 0.77) showed that there were no significant differences in the Symbiodiniaceae community of *G. fascicularis* across four seasons.

**Figure 3:**
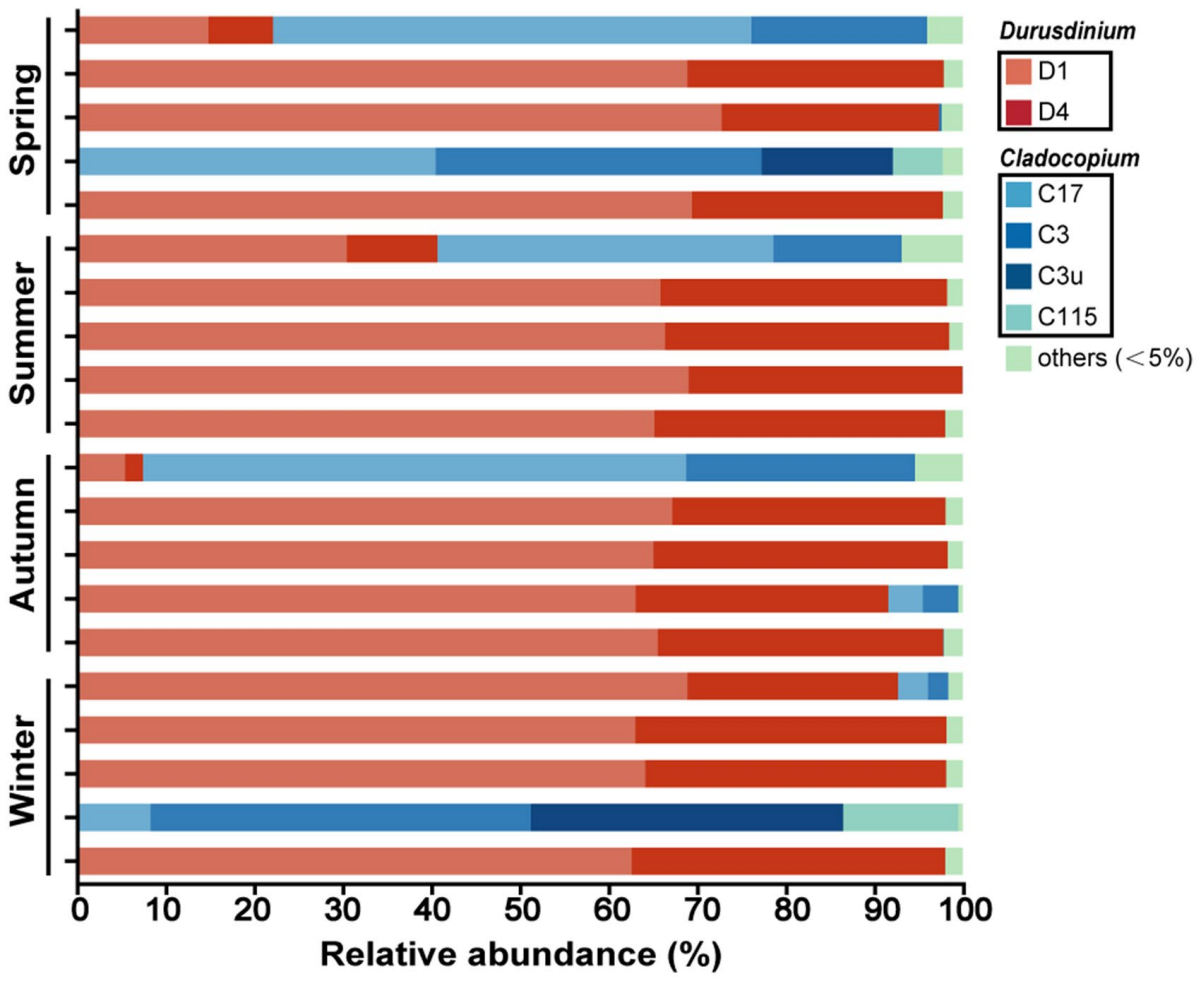
*Symbiodiniaceae* community composition in four seasons, including spring (*n* = 5), summer (*n* = 5), autumn (*n* = 5), and winter (*n* = 5). Bars indicated the relative abundance of each type. Types with relative abundance <5% were combined as others.

The composition of the Symbiodiniaceae community at the ASV level in *G. fascicularis* remained remarkably stable throughout all four seasons, primarily consisting of ASV1 (*Durusidinium* sp. D1) and ASV2 (*Durusidinium* sp. D4), with only a limited presence of *Cladocopium* sp. within an individual (Figure 3). Furthermore, a decrease in the abundance of *Cladocopium* (C17, C3, C3u, and C115) was observed from spring to summer, accompanied by an increase in the proportion of *Durusidinium* (D1 and D4). Meanwhile, the high abundance of *Durusidinium* persisted until autumn and winter, which was still predominated by *Durusidinium* sp. D1.

### 3.6 Bacterial composition, functional prediction, and correlation network

**Figure 4:**
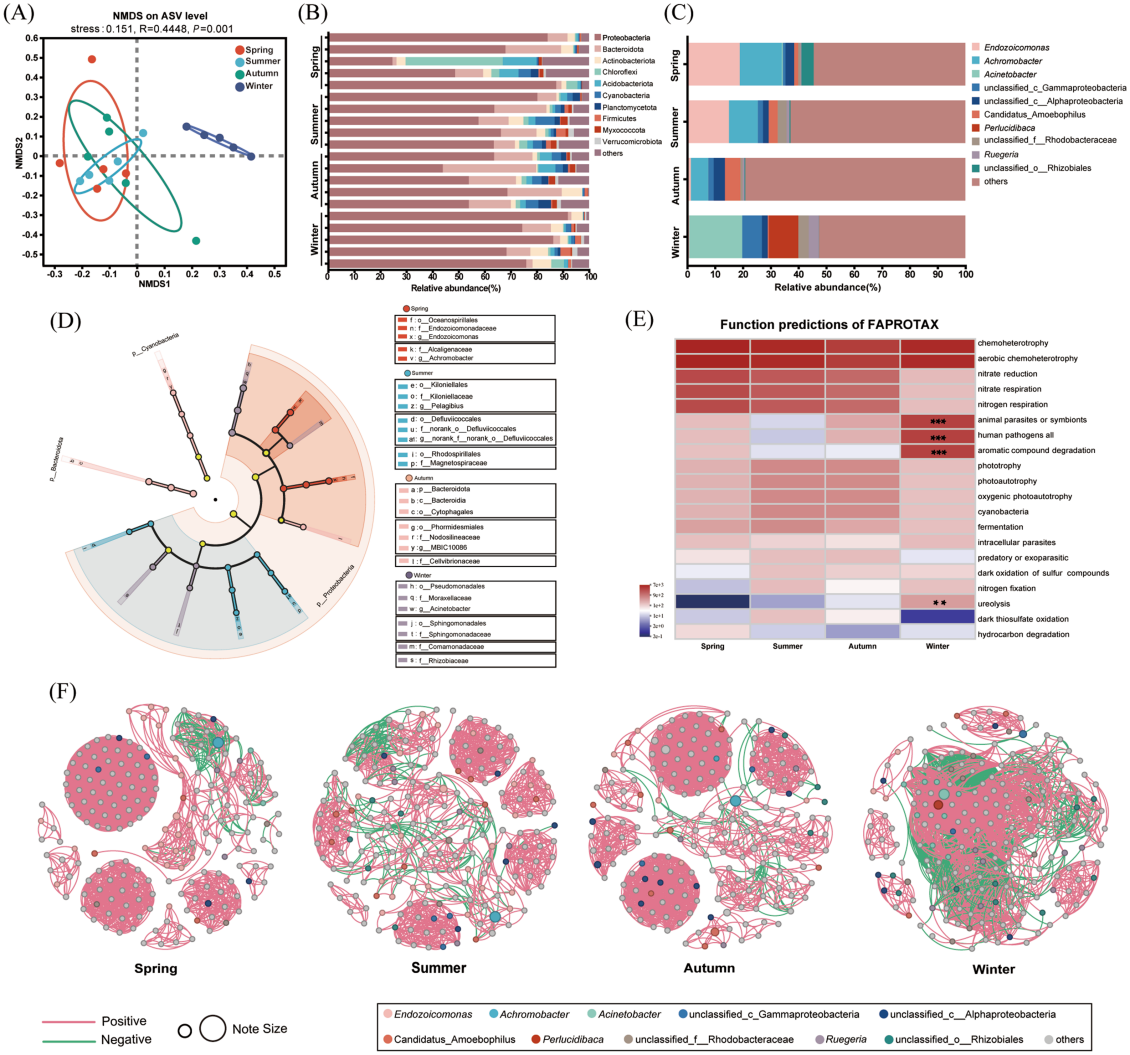
Bacterial composition, functional prediction, and correlation network. **(A)** NMDS analyses based on the Bray–Curtis distance. **(B)** The top 10 bacterial phyla are shown. **(C)** The top 10 bacterial genera are shown. **(D)** LDA discriminant analyses revealed significantly enriched distinct species from the phylum to genus level. Different colored regions represent different constituents (red: spring, blue: summer, pink: autumn, purple: winter, yellow: non-significant). The diameter of each circle was the relative abundance of the group. **(E)** FAPROTAX *analyses p*redicted the top 20 microbial ecological functions across the four seasons. ** 0.001 < *p* ≤ 0.01, ***p ≤ 0.001. **(F)** The single-factor correlation network analysis of the bacterial community at the ASV level. The nodes colored represented the genus level for bacteria, and the size of each node was proportional to the relative abundance of species.

A total of 913,949 high-quality reads were obtained from 20 samples, with an average of 45,697 reads per sample. After removing ASV sequences affiliated with the chloroplast and mitochondria, the libraries were classified into 15,463 ASVs. The coverage of the ASVs was 99.74 ± 0.001%, and the rarefaction curves showed the sequencing volume was sufficient to cover the microbial communities in all samples (Supplementary Figure S3). Furthermore, NMDS analyses based on the Bray–Curtis distance (Figure 4A, stress = 0.151, *R* = 0.4448, *p* = 0.001) and the PERMANOVA result (*F* = 1.89, *R^2^* = 0.26, *p* = 0.001) showed that the bacterial community composition of *G. fascicularis* in winter was significantly different compared to the other three seasons, yet it displays the lowest inter-group variability.

At the phylum level, Proteobacteria (56.88–79.42%), Bacteroidota (8.63–31.10%), and Actinobacteriota (3.19–5.18%) were abundant in *G. fascicularis* (Figure 4B). Proteobacteria was dominant in all four seasons, while the abundance of Bacteroidota increased specifically in autumn (Kruskal–Wallis test, *p* < 0.05). At the genus level, *G. fascicularis* was dominated by *Endozoicomonas* and *Achromobacter* in spring and summer (Figure 4C). However, their relative abundance reduced by 20.71 and 31.23%, respectively, in summer compared to spring. In autumn, *Achromobacter* became dominant (6.15%), while *Endozoicomona* decreased significantly (1.36%). *Acinetobacter* (19.03%) was the most dominant, followed by *Perlucidibaca* (10.62%) and unclassified_c_Gammaproteobacteria (7.10%) in winter, which was different from the other three seasons. LDA discriminant analyses revealed 27 significantly enriched distinct species at the phylum to genus level (Figure 4D, LDA > 4, *p* < 0.05). At the genus level, *Endozoicomonas* and *Achromobacter* have significant enrichment in spring. *Pelagibius* and g_norank_Defluviicoccales were summer difference species. MBIC10086 and *Acinetobacter* were significantly enriched in autumn and winter, respectively. Additionally, the bacterial community of *G. fascicularis* harbored a high proportion of low-abundance taxa across seasons, forming a stable foundational component. Taxonomic inspection revealed it to be a diverse assemblage of phyla, including Planctomycetota, Chloroflexi, and Acidobacteriota, alongside numerous unclassified lineages within dominant phyla like Proteobacteria, and many of these ASVs could not be classified beyond the order or family level (Supplementary Table S5).

FAPROTAX analyses predicted the top 20 microbial ecological functions across the four seasons. Ecological functions, such as animal parasites or symbionts, human pathogens all, automatic compound degradation, and ureolysis were significant positively correlated in winter (Kruskal–Wallis test, FDR-correction, *P*-adjust < 0.05, Figure 4E). The predicted results from PICRUSt2 revealed a stronger positive correlation between *Endozoicomonas* and pyruvate metabolism (Spearman, *r* = 0.548, *p* < 0.05, Supplementary Figure S4). Additionally, *Achromobacter* was positive with a two-component system and quorum sensing (Spearman, *r* = 0.632, *p* < 0.05; Spearman, *r* = 0.653, *p* < 0.05). *Acinetobacter* dominated in winter and was negative with glycolysis/gluconeogenesis, cysteine and methionine metabolism, and carbon metabolism (*p* < 0.05). The correlation network analysis at the ASV level showed that in winter, the bacterial network consisted of 199 nodes and 2,752 edges, exhibiting the highest average degree (27.658) and the lowest average path length (2.501) among all four seasons. The co-occurrence patterns showed that bacterial communities were more closely related and had a higher negative correlation (35.94%) in winter than in the other three seasons. Additionally, the core bacteria *Acinetobacter* had a negative interaction with other bacteria in winter (Figure 4F).

### 3.7 Seasonal transcriptomic responses of coral host *G. fascicularis*

A total of 17 *G. fascicularis* samples (spring, *n* = 4; summer, *n* = 4; autumn, *n* = 4; winter, *n* = 5) were subjected to transcriptome analysis, resulting in 122.11 Gb of clean data, and the clean data of each sample reached more than 6.19 Gb. Furthermore, the Q20 was > 98.31%, the Q30 was > 94.99%, and the sequencing error rate was ≤ 0.013%. The sequence comparison of clean reads of each coral sample with the reference genome (*Galaxea fascicularis*, gfas_1.0) showed that the comparison rate ranged from 32.46 to 54.23%.

**Figure 5:**
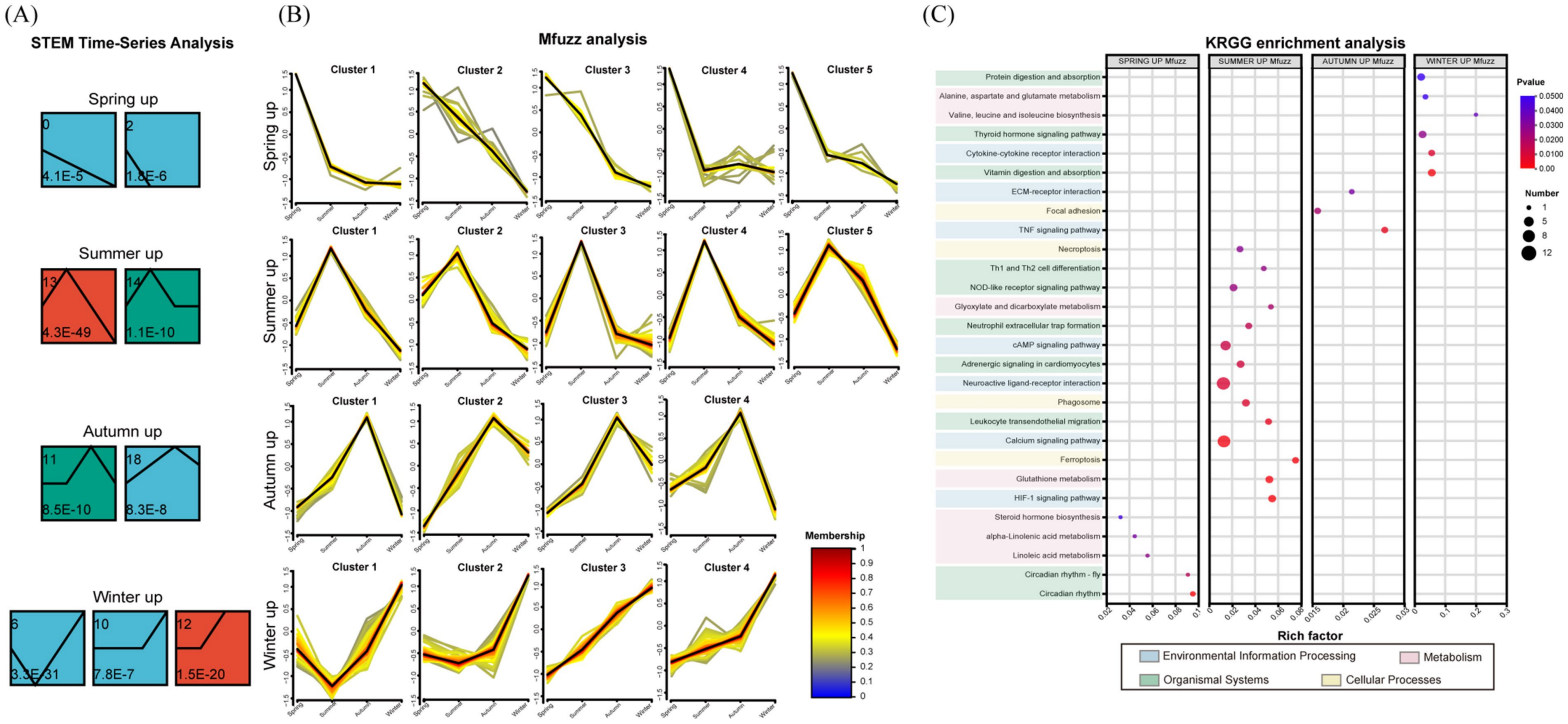
Seasonal transcriptomic responses of coral host *G. fascicularis*. **(A)** The short time-series expression miner (STEM) program classified the upregulated DEGs clusters were identified according to the temporal gene expression patterns (*P*-adjust < 0.05). **(B)** Mfuzz expression pattern clustering validated results. **(C)** KEGG enrichment analysis of up-DEGs among four seasons (*p* < 0.05).

The STEM program was used to classify 1783 DEGs (spring = 330, summer = 511, autumn = 527, winter = 415, respectively) into possible model clusters based on temporal gene expression patterns. Within these clusters, the expression patterns of the upregulated DEGs clusters (spring = 48, summer = 159, autumn = 72, winter = 209, respectively) were identified (*P*-adjust < 0.05) (Figure 5A). After Mfuzz validation, five DEGs sets were obtained in spring (23 DEGs) and summer (159 DEGs), and four DEGs sets were obtained in autumn (58 DEGs) and winter (166 DEGs) (Figure 5B). The top 20 pathway enrichment results across four seasons are shown (Figure 5C, Supplementary Tables S6, S7, *p* < 0.05). The spring transcriptome aligned with growth and reproductive preparation, and the up-DEGs set was enriched in linoleic acid metabolism, steroid hormone biosynthesis, and the circadian rhythm pathway. Key upregulated genes included PLA2G (log2FC > 1.31, *P*–adjust < 0.05), which participated in alpha-linolenic acid metabolism (conversion of phosphatidylcholine to alpha-linolenic acid) and linoleic acid metabolism (conversion of lecithin to linoleate). In addition, the core circadian rhythm genes CLOCK (2.66 < log2FC < 3.44, *P*-adjust < 0.05) and BHLHB2 (1.58 < log2FC < 2.29, *P*-adjust < 0.05), which are potential regulators of spawning timing. The summer displayed a prominent heat stress signature, with coordinated activation of signaling (HIF–1, calcium signaling, cAMP), cell death regulation (ferroptosis, phagosome, necroptosis), and immune pathways (NOD-like receptor, Th1/Th2 cell differentiation). Concordantly, accompanied by upregulation of the NOX2 gene (1.11 < log2FC < 2.46, *P*–adjust < 0.05), which participated in cellular immune and apoptosis, the GCLM gene (1.15 < log2FC < 1.45, *P*–adjust < 0.05) was involved in ferroptosis-related pathways, and the CAMK gene (2.68 < log2FC < 2.69, *P*–adjust < 0.05) was related to stress-signaling homeostasis. The autumn focused on immune repair and tissue remodeling via enriched TNF signaling, focal adhesion, and ECM–receptor interaction pathways, with key COL6A (1.12 < log2FC < 3.05, *P*-adjust<0.05) supporting extracellular matrix stabilization. Winter was characterized by metabolic reprogramming and recovery, with upregulated DEGs enriched in nutrient metabolic pathways (vitamin digestion and absorption, and amino acid synthesis). Core genes included the BMP2 (1.30 < log2FC < 2.42, *P*-adjust<0.05), the BMP7 (2.40 < log2FC < 4.80, *P*-adjust<0.05), and the SLC16A10 (1.31 < log2FC < 2.15, *P*-adjust<0.05), belonging to the calcium channel and skeleton morphogenetic proteins. These changes align with enhanced energy storage and stable autotrophic nutrition (Δ^h-z 13^C > 0) in winter.

### 3.8 Seasonal transcriptomic responses of coral Symbiodiniaceae

Following the Symbiodiniaceae community composition analyses, 75% of coral samples primarily consisted of *Durusdinium* sp. Subsequently, the 13 coral samples passed transcriptome quality control and were applied for transcriptome analyses of the Symbiodiniaceae. This resulted in 94.28 Gb of clean data being obtained, with each sample reaching more than 6.19 Gb. Furthermore, a base quality value of Q20 was > 98.31%, Q30 was > 94.99%, and the sequencing error rate was ≤ 0.013%. Each coral sample alignment with the reference genome (*Durusdinium trenchii*) ranged from 13.52 to 32.41%. DEGs profiles gained 1,008 upregulated DEGs (spring = 391, summer = 109, autumn = 145, winter = 363, respectively) and 1,046 downregulated DEGs (spring = 404, summer = 117, autumn = 222, winter = 303, respectively) (*P*-adjust < 0.05). The STEM analysis showed 254, 4, 16, and 88 significantly upregulated DEGs and 326, 23, 88, and 94 significantly downregulated DEGs in spring, summer, autumn, and winter, respectively (*p* < 0.05).

There were six upregulated pathways and four downregulated pathways enriched in the given DEGs sets (Supplementary Tables S8–S10, *p* < 0.05). The upregulated DEGs in spring were enriched in photosynthesis, ribosome, and homologous recombination pathways, while the downregulated DEGs were associated with the motor proteins pathway. In the photosynthesis pathway, the *psaC* (1.39 < log2FC < 2.69, *p* < 0.05) and *psaE* (1.01 < log2FC < 2.66, *p* < 0.05) assigned to PS I, the *psbY* (1.25 < log2FC < 1.43, *p* < 0.05) assigned to PS II, and the *petF* (1.22 < log2FC < 2.69, *p* < 0.05) encoding ferredoxin, and they were upregulated. There were no upregulated DEGs observed in summer and autumn. The HPGDS gene (5.72 < |log2FC| < 7.21, *p* < 0.05) belonged to the arachidonic acid metabolism pathway, and the glutathione metabolism pathway was downregulated in summer. In autumn, downregulated DEGs were mostly enriched in pyruvate metabolism and protein processing in the endoplasmic reticulum pathway. In winter, upregulated DEGs enriched the amino acid metabolism, including phenylalanine metabolism, tyrosine and tryptophan biosynthesis, and histidine metabolism.

## 4 Discussion

### 4.1 Spring endosymbiotic microalgae benefits for growth and reproduction

The mean seawater temperature in spring (March–May) in the northern WZZ Island was 27.73 °C ± 1.61, which is the optimal range for coral growth (Guan et al., 2015). The stable carbon isotope results indicated that the nutritional mode of *G. fascicularis* in spring was autotrophic, which is highly dependent on the photosynthesis of endosymbiotic microalgae (Tremblay et al., 2012; Nahon et al., 2013). Photosynthesis strongly correlated with coral endosymbiotic microalgal density and chlorophyll a (Yu et al., 2019). In our study, high levels of endosymbiotic microalgal density, chlorophyll a, Fv/Fm, Y(II), and *α* were observed in spring compared with summer (*p* < 0.05). Correspondingly, the photosystem genes (*psaC*, *psaE*, *psbY*, and *petF*) were upregulated in the Symbiodiniaceae transcriptome, suggesting that symbiotic microalgae efficiently carried out photosynthesis to provide energy. Among them, *petF* is reported to be a major photosynthetic ferredoxin protein in chloroplasts that can provide energy to coral Symbiodiniaceae (Lin et al., 2013). The endosymbiotic microalgae exhibited good vitality in spring and could provide abundant photosynthetic derivatives to support the coral growth.

The reproduction of corals and their annual spawning are typical examples of cyclic biological rhythms (Ayalon et al., 2021). Previous studies have shown that the spawning of *G. fascicularis* occurs in April and May each year (Wei et al., 2023). Our spring sampling time (22 April) coincides with the pre-spawning period of *G. fascicularis*. The CLOCK gene might play a role in regulating the timing of spawning (Brady et al., 2016). The upregulation of the CLOCK gene associated with circadian rhythm suggested that coral may be prepared for the regulation of spawning time. Lipid reserves for the pre-spawning period are crucial, and the lipid content of *G. fascicularis* was significantly higher in spring than in other seasons in our results. Correspondingly, upregulated PLA2G gene participated in the biosynthesis of alpha–linolenic acid and linoleate, which may contribute energy requirements of coral hosts in spring. Unsaturated fatty acids (UFA) regulate various physiological activities such as coral reproduction and cell membrane fluidity, while also playing a crucial role in coral stress resistance (Monroig et al., 2013). Yu et al. (2024) show that local *G. fascicularis* from Sanya can adapt to seasonal fluctuations by increasing polyunsaturated fatty acids, which is consistent with our findings. Similarly, cnidarian steroid biosynthesis often obtains precursors from endosymbiotic microalgae (Lu et al., 2020). Our result showed that the SULT1E1 gene (log2FC > 2.68, *p* < 0.05, encoding estrogen sulfotransferase) was upregulated in the pre-spawning period, which synthesized steroidal estrogens by using those precursors in coral hosts (Ochsenkühn et al., 2023). As a result, the upregulation of the steroid biosynthetic pathway in *G. fascicularis* in spring indicated the efficient energy conversion process from endosymbiotic microalgae to the host, particularly facilitating coral growth and development.

### 4.2 Summer heat increases the difficulties of post-spawning recovery

Before the summer sampling, the coral went through their spawning period (recorded by Jingzhao Ke from April to May 2023), which is energy-consuming. Moreover, WZZ Island experienced prolonged periods of high seawater temperatures exceeding 30 °C, potentially causing significant thermal stress on corals. Heat stress typically leads to lipid peroxidation in corals and subsequent membrane damage (Lushchak, 2011). This process coincides with reduced levels of endosymbiotic microalgae and chlorophyll a, as well as a decrease in Fv/Fm, indicating impaired photosynthetic capacity (Chen et al., 2024). Our findings showed all *G. fascicularis* colonies exhibited mild tissue paling without severe bleaching symptoms, indicating incipient stress responses consistent with partial symbiont loss. Concurrently, summer corals displayed oxidative damage, evidenced by elevated MDA and T-AOC levels. Reduced endosymbiont density, chlorophyll a content, and photosynthetic capacity (Fv/Fm, Y(II), *α*) further implied impaired autotrophy. Zhou et al. (2023), through carbon stable isotope, showed that massive corals used the switch between heterotrophy and autotrophy to obtain energy availability. δ^13^C analysis further revealed that *G. fascicularis* exhibited heterotrophy by utilizing nutrient reserves (sugars, proteins, and lipids) to compensate for the loss of phototrophic carbon.

Moreover, Li et al. (2021a, 2021b) found that Symbiodiniaceae had a later transcriptional response than coral hosts. Similar to our results, the transcriptional responses of the coral host were more active due to symbiont microalgae elimination during summer stress. The NOX2, a member of the NADPH oxidase (NOX) enzyme family, plays a pivotal role in generating reactive oxygen species (Bode et al., 2023). In our result, the NOX2 gene (1.11 < log2FC < 2.47) was upregulated in summer, which may induce the ferroptosis pathway, which is an iron-dependent oxidative cell death accompanied by lipid peroxidation and membrane damage (Kuang et al., 2020; Li et al., 2021a). The pathways associated with apoptosis, immunity, translation, replication, and repair are generally upregulated in corals during thermal stress (Li et al., 2021a, 2021b; Zhang et al., 2022). EGLN gene (2.06 < log2FC < 2.37) associated with the HIF–1 signaling pathway was upregulated after oxidative damage. The EGLN gene encodes proline hydroxylase, which releases HIF-α in response to hypoxia by scavenging damaged mitochondria to reduce reactive oxygen species (Ivan and Kaelin, 2017). Previous findings also suggest that *Acropora tenuis* is more tolerant because it can adopt HIF faster and more efficiently than sensitive corals to regulate responses under hypoxic stress (Alderdice et al., 2021). Furthermore, our result showed that the GCLM gene was upregulated in summer, which converted glutamate to GSH via the encoded glutamate–cysteine ligase (Veeravalli et al., 2011). Previous studies reported that the main mechanism to reduce reactive oxygen species is the redox capacity of glutathione (GSH), and GSH as an antioxidant can effectively inhibit ferroptosis through the GSH–GPX4 system (Rochette et al., 2022). Moreover, CAMK in the calcium signaling pathway was upregulated in our study. The interaction between the upregulation of CAMK expression and the CAMP pathway is crucial for mitochondrial biogenesis and maintaining homeostasis (Aslam and Ladilov, 2022). Increased CAMK activity phosphorylated AMPK, while the positive regulation of AMPK activity by CAMP signaling activates the CAMK2–AMPK pathway, which regulates mitochondrial homeostasis (Aslam and Ladilov, 2022; Di Benedetto et al., 2018). These research findings provide valuable guidance on optimal transplantation timing for *G. fascicularis*. It is recommended to conduct transplantation outside the coral reproduction (April to May) and the summer high-temperature period to enhance coral adaptability and survival rates.

### 4.3 Coral recovery from autumn to winter after summer heat and reproduction

The autumn sampling time was on 22 October, after the Qiongdong upwelling [QDU, July to September, recorded by Zhu et al. (2023)]. It was regarded as a potential thermal refuge to bring nutrient-cooling seawater, alleviating coral bleaching (Lee et al., 2020; Zhu et al., 2022). The seawater temperature surrounding *G. fascicularis* consistently declined in autumn and winter, indicating a potential recovery stage for corals after heat stress and reproduction. The inference was supported by the recovery of coral host energy and endosymbiotic microalgal capacity in our results, as shown by the increase in density, elevated levels of chlorophyll a, and enhanced photosynthesis. In this case, *G. fascicularis* exhibited a transition from heterotrophy in summer to autotrophy in autumn and winter. Additionally, upregulated DEGs in Symbiodiniaceae were enriched in the biosynthesis pathways of phenylalanine, phenylalanine, tyrosine and tryptophan, and histidine in winter. These synthesized amino acids can be provided into the coral host through photosynthesis (Shinzato et al., 2014).

In this study, there were few DEGs in the coral transcriptome between summer and autumn, suggesting that the coral host was still in a continuous process of immunoregulatory response to repair oxidative damage. Previous studies reported that the TNF signaling pathway, ECM–receptor interaction, and focal adhesion in the coral host were related to immune stress (Sutherland et al., 2023). In our results, SOCS3 in the TNF signaling pathway was activated, which plays a key role in immunoregulation, inflammation, apoptosis, and cell proliferation and differentiation (Cho et al., 2003). It may enhance the antimicrobial immunity by inhibiting AKT phosphorylation (Zhou et al., 2015). In addition, the extracellular matrix (ECM) has an interdependent relationship with the immune system and can participate in it by guiding the migration of immune cells (Sutherland et al., 2023). The COL6A gene was upregulated in the ECM–receptor interaction pathway and the focal adhesion, providing structural support for cells and thereby contributing to the properties of the local ECM microenvironment.

The winter-upregulated DEGs were enriched in the biosynthesis of branched-chain amino acids (leucine, isoleucine, valine), as well as vitamin and protein digestion and absorption. This finding suggested that the metabolic processes for the coral host’s energy restoration remained active in winter. Previous studies showed that all intracellular components of BMP (Bone morphogenetic proteins) signaling are present in cnidarian (Mörsdorf et al., 2024). BMP2 and BMP7 are widely recognized as the primary initiators of skeleton formation and regeneration (Salazar et al., 2016). SLC16A10, as a calcium channel protein, plays a role in regulating skeletal formation and skeletal resorption through the alteration of Ca^2+^ concentration (Lu et al., 2021). They were also upregulated. Briefly, the process of skeleton growth, along with energy storage and nutrient metabolism, was activated in winter.

### 4.4 Potential beneficial symbionts and seasonal pathogens of *G. fascicularis*

*Endozoicomonas* is often abundant in the tissues of healthy corals and recognized as a beneficial symbiont for coral hosts (Pogoreutz et al., 2022). It can degrade coral-derived steroids and Symbiodiniaceae-derived galactose (Ochsenkühn et al., 2023). Thus, the high abundance of *Endozoicomonas* in spring contributes to the stability of efficient energy conversion among the host and symbionts. However, coral-associated bacteria, such as *Endozoicomonas*, even as a core bacterium of corals, are sensitive to heat stress and often decrease during heat stress (Chan et al., 2021; Bourne et al., 2008; McDevitt-Irwin et al., 2017). It decreased in summer and autumn compared to spring, but remained dominant in summer in our study. More importantly, under thermal stress, *Endozoicomona* partially uses coral-derived cholesterol as a carbon source and converts it to the hormones testosterone and progesterone (Ochsenkühn et al., 2023). Both steroids prime the innate immune system and inhibit pathogens (Patt et al., 2018).

The opportunistic pathogenic bacteria in *Acropora pruinosa* and *Pavona decussata* increased in summer, as reported by Yu et al. (2023). McDevitt-Irwin et al. (2017) also reported that *Defluviicoccales*, as in potentially pathogenic and opportunistic bacteria, increased when corals were under stress, while we found that summer opportunistic g_*Pelagibius* and o_Defluviicoccales rose under host damage. FAPROTAX predicted that *Acinetobacter*, which concentrates in winter and was recognized as a potential pathogenic bacterium, is generally one of the opportunistic pathogens and can be vertically transmitted through mucus in corals (Leite et al., 2017; Yu et al., 2023). Further, bacterial network analyses indicated that there was a tendency toward a negative correlation in competitive niches, and they competed with *Endozoicomonas* for ecological niches. Predicted function was based on metabarcoding data, and potential broader ecological functions of the role of pathogenic bacteria in coral hosts are necessary. Notably, the winter bacterial community showed the lowest inter-sample variation, corresponding to the most stable and lowest temperatures of the year, indicating that environmental stability likely drove community homogenization (Ziegler et al., 2019). This contrasts with reports of higher winter variability in other low-latitude systems (Yang et al., 2020), emphasizing that local environmental conditions may be more significant than mere latitude labels when understanding the seasonal dynamics of coral microbiomes. Additionally, the bacterial community of *G. fascicularis* harbored a high proportion of low-abundance taxa across seasons, forming a stable foundational component. While their specific functions remain incompletely elucidated, the consistent presence of these rare taxa implies they may act as a functional diversity reservoir, potentially enhancing community resilience, and contributing to ecosystem processes (Lynch Michael and Neufeld Josh, 2015; Jousset et al., 2017).

## 5 Conclusion

As a heat-tolerant coral species in the South China Sea, *G. fascicularis* has a dual nutritional system that is dynamically cooperated by the host and endosymbiotic microalgae/bacteria when facing long-term seasonal fluctuations. During spring, autumn, and winter, it mainly relied on autotrophic photosynthesis for growth, but in summer, it turned to heterotrophic predation to maintain energy reserves. The efficiency of endosymbiotic microalgae in photosynthesis benefited growth, especially activating the unsaturated fatty acid (UFA) pathway that might have been related to lipid reserves before spawning. However, when faced with the oxidative damage caused by seasonal heat stress, *G. fascicularis* transformed trophic patterns, mobilized the antioxidant system, and implemented complex immune regulation to increase its resilience, rather than shuffling the *Durusidinium* for short-term recovery. Coral recovery occurred in autumn and winter. In autumn, it continued to regulate immunity to repair oxidative damage, while in winter, it activated processes for skeletal growth, energy storage, and metabolism. Potential pathogens of *G. fascicularis* were seen more commonly in winter than in summer, potentially competing with the existing beneficial bacterium *Endozoicomonas* for ecological niches.

## Data Availability

The datasets presented for this study can be found in China National Center for Bioinformation/Beijing Institute of Genomics, Chinese Academy of Sciences (https://ngdc.cncb.ac.cn/gsa), accession CRA019262, CRA019263, and CRA019680.

## Footnotes

1 http://www.geruisi-bio.com/

2
http://sym-its2.marinegenomics.cn/Annotation

3
http://gfas.reefgenomics.org/download/

4
http://mfuzz.sysbiolab.eu/

5
https://cloud.majorbio.com/page/tools/

6
https://coralreefwatch.noaa.gov/product/vs/gauges/hainan_island.php
