# Supplementary material for: Seasonal dynamics of Galaxea fascicularis holobiont from physiological to transcriptional responses and implications for natural resilience
Source: Front Microbiol. 2025 Dec 19;16:1707108. doi: 10.3389/fmicb.2025.1707108 (PMC12757359; doi:10.3389/fmicb.2025.1707108)
Supplement: Supplementary file 1 [file Data_Sheet_1.PDF]

frontiers

Supplementary Material

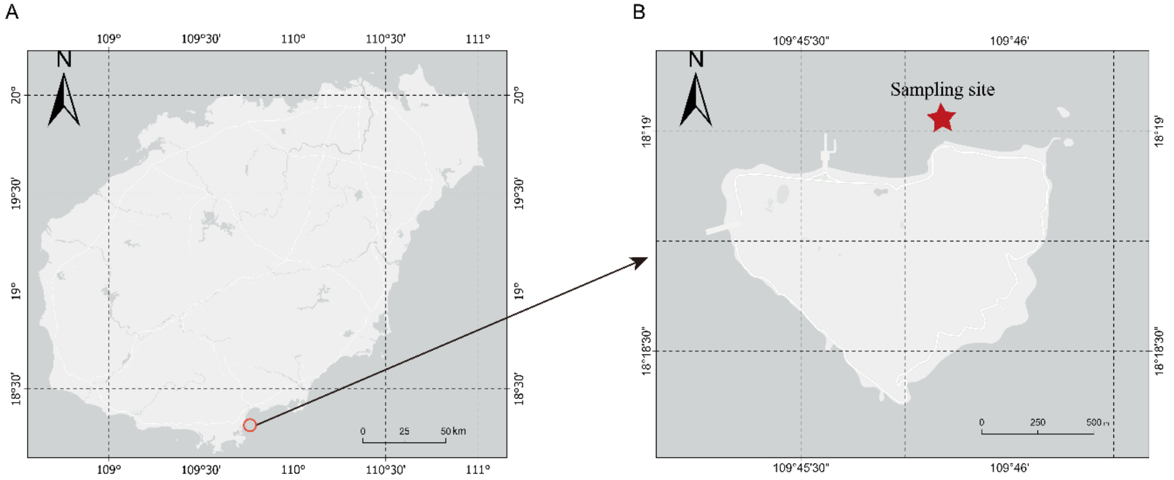

Figure S1 Map showing the geographical location of the sampling site on the northern of WZZ Island (Sanya, China).

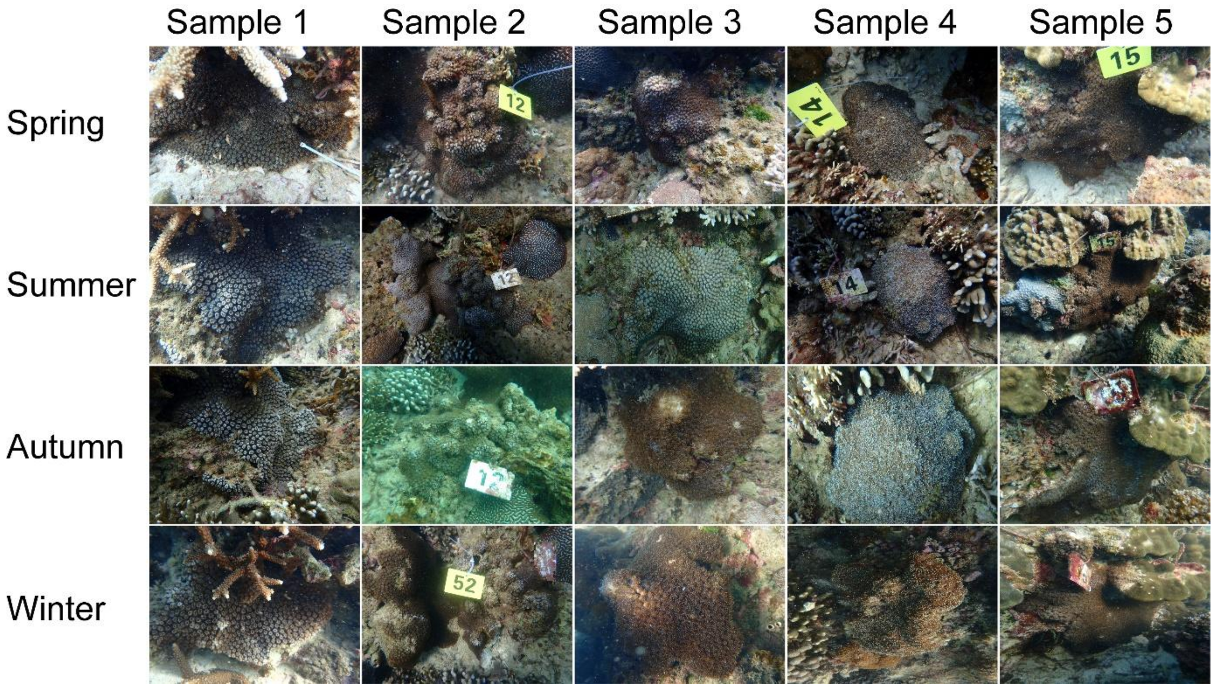

Figure S2 Morphological identification to determine five coral species as *Galaxea fascicularis* and labeled numbered cards. Notes: The labeled numbered cards got corroded by seawater in winter. So, we replaced numbers (11 - 15) with numbers (51 - 55).

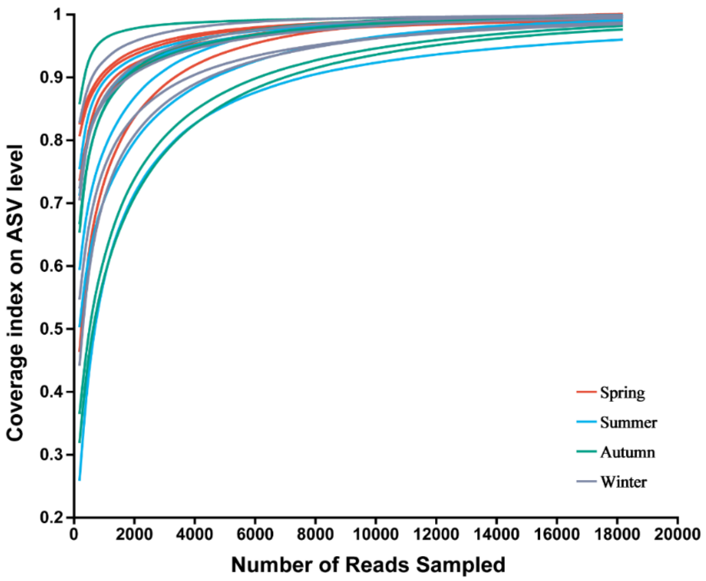

Figure S3 Rarefaction curves of microbial communities' coverage index on ASV level.

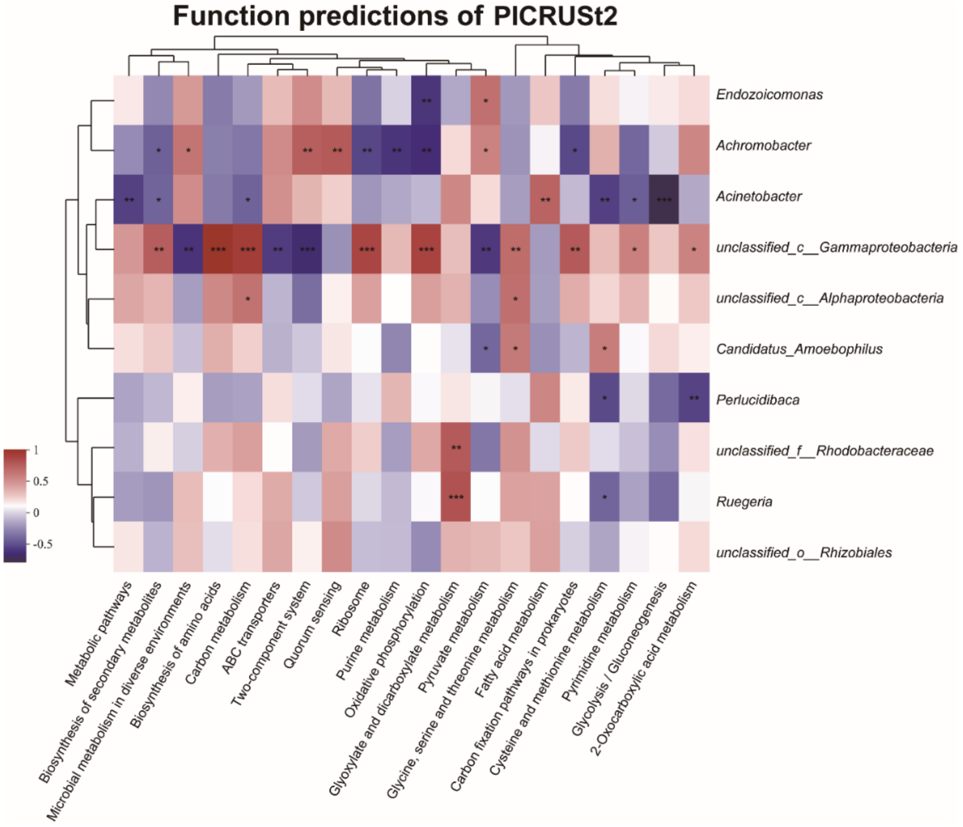

Figure S4 Top 10 coral associated bacteria function predictions based on PICRUSt2.
